# 8β-Eth­oxy­eremophil-3,7(11)-diene-8α,12;6α,15-diolide

**DOI:** 10.1107/S1600536813015729

**Published:** 2013-06-12

**Authors:** Dong-Qing Fei, Le-Le Dong, Hui-Hong Li, Zhan-Xin Zhang

**Affiliations:** a School of Pharmacy, Lanzhou University, Lanzhou 730000, People’s Republic of China

## Abstract

The title compound, C_17_H_20_O_5_, an eremophilane sesquiternoid, was isolated from the roots of *Ligularia lapathifolia*. The mol­ecule contains four fused rings of which the six-membered ring *A* adopts a half-chair conformation, the six-membered ring *B* adopts a chair conformation, the five-membered ring *C* is almost planar (r.m.s. deviation = 0.015 Å) and the five-membered ring *D* adopts an envelope conformation with the quaternary C atom as the flap. The methyl and the eth­oxy groups adopt a *syn* conformation and the *A*/*B* ring junction is *cis*-fused. No directional inter­molecular inter­actions could be identified in the crystal.

## Related literature

For further information on the isolation of the title compound, see Fei *et al.* (2007 ▶). For pucking paramaters, see: Cremer & Pople (1975 ▶); Boeyens (1978 ▶)
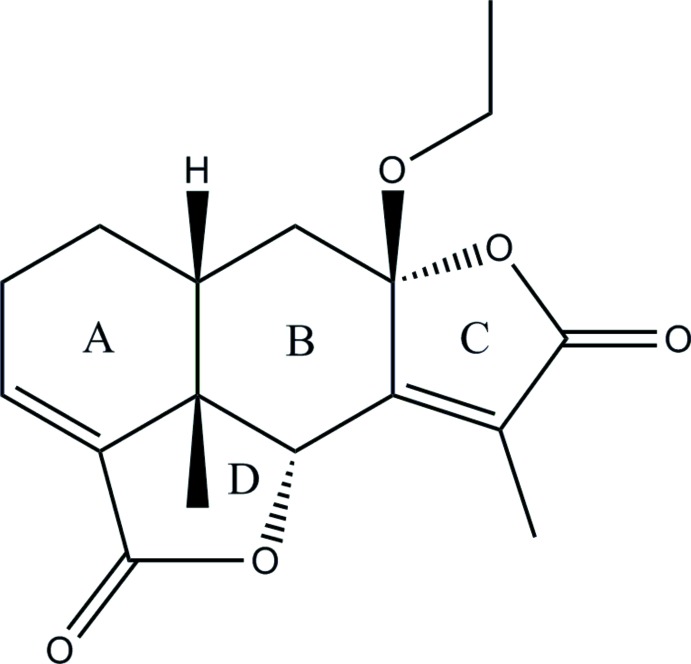


## Experimental

### 

#### Crystal data

C_17_H_20_O_5_
*M* *_r_* = 304.33
Orthorhombic, 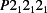

*a* = 8.4925 (2) Å
*b* = 13.0302 (4) Å
*c* = 14.1381 (9) Å
*V* = 1564.50 (12) Å^3^
*Z* = 4
Cu *K*α radiation
μ = 0.78 mm^−1^
*T* = 294 K
0.33 × 0.28 × 0.12 mm

#### Data collection

Agilent SuperNova (Dual, Cu at zero, Eos) diffractometer
Absorption correction: multi-scan (*CrysAlis PRO*; Agilent, 2013 ▶) *T*_min_ = 0.743, *T*_max_ = 1.000
14336 measured reflections
2994 independent reflections
2871 reflections with *I* > 2σ(*I*)
*R*_int_ = 0.027

#### Refinement

*R*[*F* ^2^ > 2σ(*F* ^2^)] = 0.037
*wR*(*F* ^2^) = 0.103
*S* = 1.03
2994 reflections
202 parameters
H-atom parameters constrained
Δρ_max_ = 0.13 e Å^−3^
Δρ_min_ = −0.18 e Å^−3^
Absolute structure: Flack (1983 ▶)
Flack parameter: −0.1 (2)

### 

Data collection: *CrysAlis PRO* (Agilent, 2013 ▶); cell refinement: *CrysAlis PRO*; data reduction: *CrysAlis PRO*; program(s) used to solve structure: *SUPERFLIP* (Palatinus & Chapuis, 2007 ▶); program(s) used to refine structure: *SHELXL97* (Sheldrick, 2008 ▶); molecular graphics: *OLEX2* (Dolomanov *et al.*, 2009 ▶); software used to prepare material for publication: *OLEX2*.

## Supplementary Material

Crystal structure: contains datablock(s) global. DOI: 10.1107/S1600536813015729/hb7083sup1.cif

Additional supplementary materials:  crystallographic information; 3D view; checkCIF report

## Figures and Tables

**Fig. 1.**
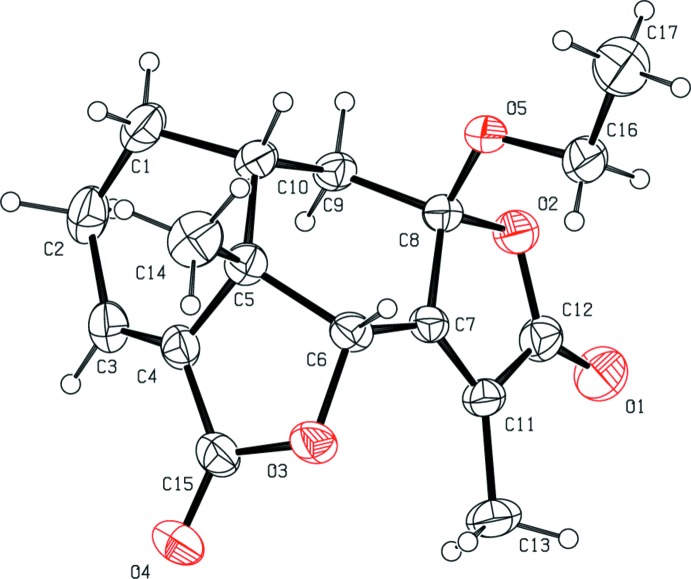
The molecular structure of the title compound, showing displacement ellipsoids at the 30% probability level.

## supplementary crystallographic information

### Comment

The title compound, 8*β*-ethoxyeremophil-3,7(11)-diene-8*α*,12(6*α*,15)-diolide (Fig. 1), was isolated from the roots of *Ligularia lapathifolia* (Fei *et al.*, 2007). The title compound is composed of four rings, two six-membered and two five-menbered. The six-membered ring *A* adopt a half-chair conformation, which has pucking paramaters (Cremer & Pople, 1975; Boeyens, 1978) Q = 0.4766 (19) Å, θ = 130.0 (2)°, φ = 133.8 (3)°. The six-membered ring *B* adopt a chair conformation with pucking paramaters Q = 0.5073 (17) Å, θ = 22.08 (19)°, φ = 205.7 (5)°. The five-membered ring *C* is almost planar with a mean torsion angle of 1.50 (8)°. The five-membered ring *D* adopt an envelope conformation. The A/B ring junction is *cis*-fused (torsion angle C14—C5—C10—H10 = 43°).

### Experimental

The air-dried roots of *L. lapathifolia* (3.6 kg) were powdered and extracted with petroleum ether -diethyl ether-acetone (1:1:1) three times successively at room temperature. The combined extracts were concentrated in vacuum to obtain a residue (270 g), which was subjected to silica gel CC and eluted with petroleum ether-acetone (50:1, 20:1, 8:1, 5:1, 2:1, 1:1, 0:1). On the basis of differences in composition indicated by TLC, seven crude fractions (A—G) were obtained. Fraction B was further fractionated on a silica gel column using petroleum ether-acetone (30:1, 10:1, 5:1, 2:1) to give four crude fractions (B1—B4). Fraction B3 was further fractionated on a silica gel column using petroleum ether-acetone (20:1, 8:1, 2:1) to obtain the pure title compound. Colourless blocks were obtained by slow evaporation of a methanol solution at room terperature.

### Refinement

All H atoms attached to C atoms were fixed geometrically and treated as riding with C—H = 0.96 Å (methyl), 0.97 Å (methylene), 0.98 Å (methine) and 0.93 Å (C=CH) with *U*_iso_(H) = 1.2*U*_ep_(C) or *U*_iso_(H) = 1.5*U*eq(C_methyl_). The Flack parameter tentatively indicates the absolute structure.

### Crystal data

**Table:**

C_17_H_20_O_5_	*D*_x_ = 1.292 Mg m^−^^3^
*M**_r_* = 304.33	Cu *K*α radiation, λ = 1.5418 Å
Orthorhombic, *P*2_1_2_1_2_1_	Cell parameters from 9242 reflections
*a* = 8.4925 (2) Å	θ = 4.6–70.7°
*b* = 13.0302 (4) Å	µ = 0.78 mm^−^^1^
*c* = 14.1381 (9) Å	*T* = 294 K
*V* = 1564.50 (12) Å^3^	Block, colourless
*Z* = 4	0.33 × 0.28 × 0.12 mm
*F*(000) = 648	

### Data collection

**Table:**

Agilent SuperNova (Dual, Cu at zero, Eos) diffractometer	2994 independent reflections
Radiation source: SuperNova (Cu) X-ray Source	2871 reflections with *I* > 2σ(*I*)
Mirror monochromator	*R*_int_ = 0.027
Detector resolution: 16.0733 pixels mm^-1^	θ_max_ = 70.8°, θ_min_ = 4.6°
ω scans	*h* = −10→9
Absorption correction: multi-scan (*CrysAlis PRO*; Agilent, 2013)	*k* = −15→15
*T*_min_ = 0.743, *T*_max_ = 1.000	*l* = −16→17
14336 measured reflections	

### Refinement

**Table:**

Refinement on *F*^2^	Secondary atom site location: difference Fourier map
Least-squares matrix: full	Hydrogen site location: inferred from neighbouring sites
*R*[*F*^2^ > 2σ(*F*^2^)] = 0.037	H-atom parameters constrained
*wR*(*F*^2^) = 0.103	*w* = 1/[σ^2^(*F*_o_^2^) + (0.0636*P*)^2^ + 0.1574*P*] where *P* = (*F*_o_^2^ + 2*F*_c_^2^)/3
*S* = 1.03	(Δ/σ)_max_ = 0.003
2994 reflections	Δρ_max_ = 0.13 e Å^−^^3^
202 parameters	Δρ_min_ = −0.18 e Å^−^^3^
0 restraints	Absolute structure: Flack (1983), 000 Friedel pairs
Primary atom site location: iterative	Flack parameter: −0.1 (2)

### Special details

**Table:**

Experimental. CrysAlisPro, Agilent Technologies, Version 1.171.36.28 (release 01-02-2013 CrysAlis171 .NET) (compiled Feb 1 2013,16:14:44) Empirical absorption correction using spherical harmonics, implemented in SCALE3 ABSPACK scaling algorithm.
Geometry. All e.s.d.'s (except the e.s.d. in the dihedral angle between two l.s. planes) are estimated using the full covariance matrix. The cell e.s.d.'s are taken into account individually in the estimation of e.s.d.'s in distances, angles and torsion angles; correlations between e.s.d.'s in cell parameters are only used when they are defined by crystal symmetry. An approximate (isotropic) treatment of cell e.s.d.'s is used for estimating e.s.d.'s involving l.s. planes.
Refinement. Refinement of *F*^2^ against ALL reflections. The weighted *R*-factor *wR* and goodness of fit *S* are based on *F*^2^, conventional *R*-factors *R* are based on *F*, with *F* set to zero for negative *F*^2^. The threshold expression of *F*^2^ > σ(*F*^2^) is used only for calculating *R*-factors(gt) *etc*. and is not relevant to the choice of reflections for refinement. *R*-factors based on *F*^2^ are statistically about twice as large as those based on *F*, and *R*- factors based on ALL data will be even larger.

### Fractional atomic coordinates and isotropic or equivalent isotropic displacement parameters (Å^2^)

**Table:**

	*x*	*y*	*z*	*U*_iso_*/*U*_eq_	
O1	0.9183 (2)	0.64050 (12)	0.11330 (10)	0.0794 (4)	
O2	0.79269 (15)	0.60175 (8)	0.24732 (9)	0.0556 (3)	
O3	0.82873 (14)	0.24550 (10)	0.20230 (9)	0.0580 (3)	
O4	0.6780 (2)	0.17448 (13)	0.09057 (11)	0.0839 (5)	
O5	0.81102 (14)	0.52062 (10)	0.39285 (7)	0.0514 (3)	
C1	0.3694 (2)	0.36787 (16)	0.35509 (16)	0.0642 (5)	
H1A	0.3487	0.3085	0.3945	0.077*	
H1B	0.3133	0.4257	0.3822	0.077*	
C2	0.3051 (2)	0.34733 (16)	0.25617 (17)	0.0678 (5)	
H2A	0.2071	0.3096	0.2617	0.081*	
H2B	0.2818	0.4124	0.2260	0.081*	
C3	0.4146 (2)	0.28847 (14)	0.19487 (14)	0.0582 (4)	
H3	0.3795	0.2650	0.1365	0.070*	
C4	0.5619 (2)	0.26851 (12)	0.22151 (12)	0.0484 (4)	
C5	0.63488 (19)	0.29910 (12)	0.31377 (12)	0.0467 (4)	
C6	0.80727 (18)	0.31837 (12)	0.27873 (11)	0.0457 (4)	
H6	0.8817	0.3034	0.3299	0.055*	
C7	0.83272 (17)	0.42502 (11)	0.24365 (11)	0.0416 (3)	
C8	0.75684 (19)	0.50977 (12)	0.30066 (11)	0.0436 (3)	
C9	0.58186 (19)	0.49238 (12)	0.30652 (12)	0.0460 (4)	
H9A	0.5376	0.4907	0.2433	0.055*	
H9B	0.5331	0.5486	0.3407	0.055*	
C10	0.5468 (2)	0.39090 (12)	0.35713 (12)	0.0481 (4)	
H10	0.5794	0.3977	0.4233	0.058*	
C11	0.89795 (19)	0.46356 (14)	0.16573 (11)	0.0485 (4)	
C12	0.8750 (2)	0.57663 (15)	0.16860 (12)	0.0551 (4)	
C13	0.9784 (2)	0.4157 (2)	0.08268 (13)	0.0680 (5)	
H13A	1.0069	0.3463	0.0978	0.102*	
H13B	1.0715	0.4541	0.0676	0.102*	
H13C	0.9085	0.4159	0.0293	0.102*	
C14	0.6368 (3)	0.20772 (17)	0.38296 (16)	0.0726 (6)	
H14A	0.6874	0.2279	0.4408	0.109*	
H14B	0.6935	0.1516	0.3551	0.109*	
H14C	0.5307	0.1867	0.3961	0.109*	
C15	0.6863 (2)	0.22373 (14)	0.16241 (14)	0.0575 (4)	
C16	0.9771 (2)	0.5300 (2)	0.40545 (14)	0.0762 (7)	
H16A	1.0163	0.5873	0.3686	0.091*	
H16B	1.0290	0.4681	0.3835	0.091*	
C17	1.0122 (3)	0.5461 (3)	0.50483 (17)	0.0970 (9)	
H17A	1.1237	0.5406	0.5148	0.146*	
H17B	0.9588	0.4953	0.5420	0.146*	
H17C	0.9773	0.6133	0.5234	0.146*	

### Atomic displacement parameters (Å^2^)

**Table:**

	*U*^11^	*U*^22^	*U*^33^	*U*^12^	*U*^13^	*U*^23^
O1	0.0966 (11)	0.0798 (10)	0.0619 (8)	−0.0179 (9)	0.0110 (8)	0.0133 (8)
O2	0.0652 (7)	0.0444 (6)	0.0572 (7)	−0.0037 (5)	0.0106 (6)	−0.0012 (5)
O3	0.0483 (6)	0.0539 (6)	0.0718 (7)	0.0118 (5)	−0.0004 (6)	−0.0224 (6)
O4	0.0807 (10)	0.0882 (11)	0.0828 (10)	−0.0103 (9)	0.0044 (8)	−0.0454 (9)
O5	0.0490 (6)	0.0629 (7)	0.0422 (6)	−0.0064 (5)	0.0061 (5)	−0.0128 (5)
C1	0.0469 (9)	0.0657 (12)	0.0800 (13)	−0.0003 (9)	0.0186 (9)	0.0034 (10)
C2	0.0406 (8)	0.0616 (10)	0.1014 (15)	−0.0028 (8)	0.0023 (10)	0.0036 (11)
C3	0.0480 (9)	0.0559 (9)	0.0707 (11)	−0.0084 (7)	−0.0062 (8)	−0.0015 (9)
C4	0.0457 (8)	0.0414 (8)	0.0580 (9)	−0.0049 (6)	−0.0002 (7)	−0.0044 (7)
C5	0.0457 (8)	0.0435 (8)	0.0510 (9)	0.0038 (6)	0.0009 (7)	0.0012 (7)
C6	0.0404 (7)	0.0469 (8)	0.0497 (8)	0.0094 (6)	−0.0042 (7)	−0.0087 (7)
C7	0.0349 (7)	0.0486 (8)	0.0412 (7)	0.0026 (6)	−0.0023 (6)	−0.0095 (6)
C8	0.0452 (8)	0.0428 (8)	0.0427 (8)	−0.0009 (6)	0.0051 (6)	−0.0042 (6)
C9	0.0431 (8)	0.0442 (8)	0.0508 (8)	0.0066 (6)	0.0069 (7)	−0.0044 (7)
C10	0.0462 (8)	0.0507 (9)	0.0474 (8)	0.0020 (7)	0.0096 (7)	−0.0004 (7)
C11	0.0405 (8)	0.0662 (10)	0.0387 (8)	0.0000 (7)	0.0016 (6)	−0.0077 (7)
C12	0.0544 (10)	0.0647 (10)	0.0462 (9)	−0.0088 (8)	0.0015 (7)	0.0037 (8)
C13	0.0609 (11)	0.0976 (15)	0.0454 (9)	0.0077 (11)	0.0106 (8)	−0.0129 (10)
C14	0.0833 (14)	0.0608 (12)	0.0738 (12)	0.0049 (10)	0.0043 (11)	0.0214 (10)
C15	0.0575 (10)	0.0494 (9)	0.0655 (10)	−0.0025 (8)	−0.0012 (9)	−0.0146 (8)
C16	0.0539 (11)	0.1208 (19)	0.0537 (11)	−0.0224 (12)	0.0009 (9)	−0.0185 (12)
C17	0.0844 (17)	0.147 (3)	0.0600 (13)	−0.0186 (16)	−0.0166 (11)	−0.0009 (15)

### Geometric parameters (Å, º)

**Table:**

O1—C12	1.200 (2)	C6—C7	1.491 (2)
O2—C8	1.4484 (19)	C7—C8	1.511 (2)
O2—C12	1.354 (2)	C7—C11	1.331 (2)
O3—C6	1.4501 (18)	C8—C9	1.506 (2)
O3—C15	1.364 (2)	C9—H9A	0.9700
O4—C15	1.204 (2)	C9—H9B	0.9700
O5—C8	1.3894 (19)	C9—C10	1.533 (2)
O5—C16	1.427 (2)	C10—H10	0.9800
C1—H1A	0.9700	C11—C12	1.487 (3)
C1—H1B	0.9700	C11—C13	1.495 (2)
C1—C2	1.525 (3)	C13—H13A	0.9600
C1—C10	1.536 (2)	C13—H13B	0.9600
C2—H2A	0.9700	C13—H13C	0.9600
C2—H2B	0.9700	C14—H14A	0.9600
C2—C3	1.485 (3)	C14—H14B	0.9600
C3—H3	0.9300	C14—H14C	0.9600
C3—C4	1.332 (3)	C16—H16A	0.9700
C4—C5	1.498 (2)	C16—H16B	0.9700
C4—C15	1.468 (3)	C16—C17	1.452 (3)
C5—C6	1.566 (2)	C17—H17A	0.9600
C5—C10	1.538 (2)	C17—H17B	0.9600
C5—C14	1.541 (2)	C17—H17C	0.9600
C6—H6	0.9800		
			
C12—O2—C8	109.65 (12)	C8—C9—C10	110.33 (13)
C15—O3—C6	109.44 (12)	H9A—C9—H9B	108.1
C8—O5—C16	116.95 (13)	C10—C9—H9A	109.6
H1A—C1—H1B	107.7	C10—C9—H9B	109.6
C2—C1—H1A	108.8	C1—C10—C5	108.51 (14)
C2—C1—H1B	108.8	C1—C10—H10	108.2
C2—C1—C10	113.74 (15)	C5—C10—H10	108.2
C10—C1—H1A	108.8	C9—C10—C1	110.51 (14)
C10—C1—H1B	108.8	C9—C10—C5	112.97 (13)
C1—C2—H2A	108.8	C9—C10—H10	108.2
C1—C2—H2B	108.8	C7—C11—C12	107.26 (14)
H2A—C2—H2B	107.7	C7—C11—C13	133.06 (18)
C3—C2—C1	113.69 (16)	C12—C11—C13	119.65 (17)
C3—C2—H2A	108.8	O1—C12—O2	121.74 (18)
C3—C2—H2B	108.8	O1—C12—C11	129.02 (18)
C2—C3—H3	119.2	O2—C12—C11	109.25 (14)
C4—C3—C2	121.60 (18)	C11—C13—H13A	109.5
C4—C3—H3	119.2	C11—C13—H13B	109.5
C3—C4—C5	125.64 (16)	C11—C13—H13C	109.5
C3—C4—C15	126.34 (18)	H13A—C13—H13B	109.5
C15—C4—C5	107.69 (15)	H13A—C13—H13C	109.5
C4—C5—C6	98.85 (13)	H13B—C13—H13C	109.5
C4—C5—C10	110.65 (14)	C5—C14—H14A	109.5
C4—C5—C14	110.58 (15)	C5—C14—H14B	109.5
C10—C5—C6	117.14 (13)	C5—C14—H14C	109.5
C10—C5—C14	110.67 (15)	H14A—C14—H14B	109.5
C14—C5—C6	108.35 (14)	H14A—C14—H14C	109.5
O3—C6—C5	104.38 (13)	H14B—C14—H14C	109.5
O3—C6—H6	109.7	O3—C15—C4	108.67 (14)
O3—C6—C7	110.13 (12)	O4—C15—O3	120.78 (18)
C5—C6—H6	109.7	O4—C15—C4	130.56 (19)
C7—C6—C5	112.97 (12)	O5—C16—H16A	109.7
C7—C6—H6	109.7	O5—C16—H16B	109.7
C6—C7—C8	116.21 (13)	O5—C16—C17	109.64 (19)
C11—C7—C6	133.40 (14)	H16A—C16—H16B	108.2
C11—C7—C8	110.06 (14)	C17—C16—H16A	109.7
O2—C8—C7	103.73 (12)	C17—C16—H16B	109.7
O2—C8—C9	111.14 (14)	C16—C17—H17A	109.5
O5—C8—O2	109.54 (13)	C16—C17—H17B	109.5
O5—C8—C7	115.69 (14)	C16—C17—H17C	109.5
O5—C8—C9	106.89 (13)	H17A—C17—H17B	109.5
C9—C8—C7	109.89 (13)	H17A—C17—H17C	109.5
C8—C9—H9A	109.6	H17B—C17—H17C	109.5
C8—C9—H9B	109.6		
			
O2—C8—C9—C10	176.04 (12)	C7—C11—C12—O1	178.38 (19)
O3—C6—C7—C8	157.37 (13)	C7—C11—C12—O2	−1.6 (2)
O3—C6—C7—C11	−15.2 (2)	C8—O2—C12—O1	−179.84 (17)
O5—C8—C9—C10	−64.47 (16)	C8—O2—C12—C11	0.15 (19)
C1—C2—C3—C4	−8.3 (3)	C8—O5—C16—C17	176.9 (2)
C2—C1—C10—C5	−58.3 (2)	C8—C7—C11—C12	2.34 (18)
C2—C1—C10—C9	66.0 (2)	C8—C7—C11—C13	−175.70 (18)
C2—C3—C4—C5	1.3 (3)	C8—C9—C10—C1	−174.70 (14)
C2—C3—C4—C15	−171.17 (17)	C8—C9—C10—C5	−52.92 (19)
C3—C4—C5—C6	−146.24 (17)	C10—C1—C2—C3	37.6 (2)
C3—C4—C5—C10	−22.7 (2)	C10—C5—C6—O3	−151.01 (14)
C3—C4—C5—C14	100.3 (2)	C10—C5—C6—C7	−31.4 (2)
C3—C4—C15—O3	160.45 (17)	C11—C7—C8—O2	−2.24 (17)
C3—C4—C15—O4	−20.1 (3)	C11—C7—C8—O5	−122.21 (15)
C4—C5—C6—O3	−32.27 (14)	C11—C7—C8—C9	116.66 (15)
C4—C5—C6—C7	87.37 (15)	C12—O2—C8—O5	125.24 (14)
C4—C5—C10—C1	48.68 (18)	C12—O2—C8—C7	1.17 (17)
C4—C5—C10—C9	−74.22 (17)	C12—O2—C8—C9	−116.86 (15)
C5—C4—C15—O3	−13.11 (19)	C13—C11—C12—O1	−3.3 (3)
C5—C4—C15—O4	166.4 (2)	C13—C11—C12—O2	176.75 (15)
C5—C6—C7—C8	41.10 (18)	C14—C5—C6—O3	82.97 (16)
C5—C6—C7—C11	−131.49 (18)	C14—C5—C6—C7	−157.39 (14)
C6—O3—C15—O4	171.11 (18)	C14—C5—C10—C1	−74.25 (19)
C6—O3—C15—C4	−9.36 (19)	C14—C5—C10—C9	162.84 (15)
C6—C5—C10—C1	160.89 (15)	C15—O3—C6—C5	26.98 (17)
C6—C5—C10—C9	38.0 (2)	C15—O3—C6—C7	−94.55 (16)
C6—C7—C8—O2	−176.51 (13)	C15—C4—C5—C6	27.38 (16)
C6—C7—C8—O5	63.51 (18)	C15—C4—C5—C10	150.88 (14)
C6—C7—C8—C9	−57.62 (18)	C15—C4—C5—C14	−86.13 (17)
C6—C7—C11—C12	175.26 (16)	C16—O5—C8—O2	−64.2 (2)
C6—C7—C11—C13	−2.8 (3)	C16—O5—C8—C7	52.5 (2)
C7—C8—C9—C10	61.80 (17)	C16—O5—C8—C9	175.24 (17)
